# Eadyn’s predictive potential: expanding horizons in vasopressor weaning and hemodynamic management

**DOI:** 10.1186/s13613-024-01413-z

**Published:** 2024-11-26

**Authors:** Jorge Iván Alvarado-Sánchez, Sergio Salazar-Ruiz, Juan Daniel Caicedo-Ruiz, Yenny Rocío Cárdenas-Bolívar, Fredy Leonardo Carreño-Hernández, Andrés Felipe Mora-Salamanca, Carlos Santacruz-Herrera

**Affiliations:** 1 Department of Critical Medicine and Intensive Care, Fundación Santa Fe de Bogotá, Bogota, Colombia; 2 Fundación Valle de Lili, Cali, Colombia; 3 Faculty of Medicine, Universidad de Los Andes, Bogotá, Colombia

Dear Editor,

We appreciate the thoughtful feedback provided by Passos et al. regarding our systematic review, *“Predictive Value of Dynamic Arterial Elastance for Vasopressor Withdrawal: A Systematic Review and Meta-analysis.”* [Letter reference]. We welcome the opportunity to respond to their insightful comments and address specific points raised in their letter.

Eadyn is a promising tool for predicting which patients are unlikely to experience a drop in mean arterial pressure (MAP) during the vasopressor weaning [1, 2]. Therefore, this hemodynamic variable could be a practical approach to understanding patient readiness for vasopressor reduction, as highlighted in our review.

We acknowledge that the limited number of studies included—only five studies with a total of 183 patients—may impact the generalizability of our findings. Our study was not restricted solely to patients with septic shock; rather, our aim was to evaluate Eadyn’s predictive utility across all critically ill patients spectrum, allowing for any type of shock to be included. However, given the current body of literature, most studies available for inclusion were conducted in septic shock patients. We agree that further studies exploring Eadyn’s predictive utility in diverse shock types—such as neurogenic, hypovolemic, and cardiogenic shock—as well as in scenarios involving vasopressors other than norepinephrine and spontaneous ventilation [3], are required. To address this, we are currently conducting studies and establishing protocols to explore these additional contexts. We also appreciate the suggestion to investigate other vasopressors like vasopressin, which could further extend our evaluation of Eadyn in varied hemodynamic scenarios.

We concur with the authors’ observation that variability in Eadyn measurement methods across studies could introduce bias. However, with the current volume of data available, subgroup analyses and meta-regression analyses were exploratory. Many statistical analyses require a minimum of 10 studies (e.g., funnel plots) to yield reliable results, and our meta-analysis faced the limitation of an insufficient number of studies in the existing literature. We hope that future research will work toward standardizing Eadyn measurement techniques to reduce methodological variability, thereby increasing the robustness of conclusions drawn from such studies.

Passos et al. also noted the potential benefit of assessing outcomes beyond MAP reduction. In our ongoing randomized controlled trial comparing Eadyn-guided vasopressor weaning against the traditional MAP-based approach in septic shock patients [4], we are measuring additional outcomes such as the intensive care unit (ICU) length of stay, weaning duration, and mortality. These variables will provide a more comprehensive understanding of Eadyn’s impact on patient outcomes and ICU resources, thereby clarifying its effectiveness in guiding vasopressor management [4].

Finally, we acknowledge that factors such as heart rate variability and arterial stiffness may influence Eadyn’s reliability. Indeed, the association between arterial stiffness and Eadyn has been demonstrated [5], as variations in arterial compliance can affect pulse pressure variation. Notably, in patients receiving norepinephrine, arterial compliance tends to remain stable and is not altered by fluid expansion [6]. For this reason, Eadyn can predict MAP increases in critically ill patients, particularly in the context of norepinephrine use [7]. We agree that the dynamic relationship between Eadyn and fluid responsiveness adds complexity to its interpretation, and future studies could benefit from integrating fluid responsiveness assessments to further refine Eadyn’s predictive capabilities for vasopressor withdrawal.

In conclusion, we thank Passos et al. for their constructive feedback. We are committed to advancing research in this field. Understanding Eadyn’s clinical applicability, determining clear cut-off points and designing reproducible methods to estimate it are key to establishing its utility as a clinical tool for vasopressor management. We appreciate the opportunity to discuss our publication and we are open to continue the dialogue in this area of critical care.

Sincerely,

## Data Availability

Not applicable.

## Abbreviations

Eadyn: Dynamic arterial elastance
MAP: Mean arterial pressure
ICU: Intensive care unit
